# Analysis of the Broth from Bones

**Published:** 1804-10-01

**Authors:** 


### Analysis of the Broth from Bones, by M. Richard and Son.

The experiments made on the analysis of the broth from bones were published by order of the Prefect of the Department des Bouches du Rhone, and are as follow.

Exp. 1. After we had procured five pounds four ounces of bones from oxen, and freed them from the cartilagineous and tendinous parts, we reduced them to a paste by strong trituration, which was boiled for about five hours with double its weight of water in an iron pot. The broth being filtrated and cooled, two pounds four ounces of jelly were obtained. The residuum was found to be four pounds, which being again subjected to decoction with double its quantity of water, yielded two pounds one ounce of jelly. The remaining bones weighed about four pounds; they were submitted to a new decoction, and the jelly thus obtained, weighed nine ounces. Lastly, we caused the pieces of bones that remained on the filtrum, to undergo another decoction, after they had been previously triturated, and we obtained eleven ounces more of jelly. Thus five pounds of bones produced near the same quantity of jelly.

Exp. 2. Having been thus assured that the bony substance furnished a quantity of jelly sufficient for being introduced with advantage into hospitals, we thought it proper to undertake a chemical examination of this substance. With this view, a portion of jelly was dissolved in a sufficient quantity of water on a sand bath, whereby broth of a greyish colour, of rather an unpleasant smell, but not disagreeable taste, was obtained. Added to the syrupus violarum, it had no action on the blue colour; and mixed with alkali, no effervescence was perceived.

Exp. 3. Being thus convinced that no acid was contained in the above broth, it remained to examine the nature of the different salts that might be present in it, which we could not doubt were the elements of the osseous texture, consisting of a saline calcareous substance and a gelatinous matter. The reagency which we employed for this purpose, were the acidulous oxalat of pot-ash dissolved in water, which being added to a portion of the broth, occasioned no precipitation: nitrate of mercury troubled the liquor but little, which however made us presume that it contained some muriatic salts. Ammonia did not produce any change, neither did a solution of fixed alkali. Concentrated acid had likewise no action on the broth, except that it became a little turbid by the addition of concentrated sulphuric acid. These experiments led us to conclude, that if there exists a saline substance, it cannot be phosphat of lime, which would have been discovered by the acidulous oxalat of pot-ash. Turbidness occasioned by the addition of sulphuric acid and of nitrate of mercury caused us, however, to suspect the presence of muriatic salts; to examine which, we undertook

Exp. 4. Part of the jelly submitted to distillation in a glass retort, to which was annexed a recipient, yielded an insipid inodorous phlegm; but the fire being increased, the matter puffed up and blackened; it exhaled a fetid smell. The interior of the retort was filled with thick white fumes, and a second phlegm, which was mostly alkaline, passed into the retort; soon after an oily matter, of an empyreumatic smell, appeared on the surface, and a slight saline crust was formed on the sides of the recipient, which being detached, proved to be carbonat of ammonia. The residuum remaining in the retort was a coaly matter, which being incinerated, appeared to be muriat of soda and phosphat of soda. The following proceeding confirmed our opinion. This saline matter was dissolved, evaporated, and crystallized; and the greatest part was muriat of soda, which appeared from its decrepitation, easy solubility, and the known taste of common salt. With respect to to the phosphat of soda, it perfectly dissolved in water; it changed the colour of syrupus violarum into green; and exposed to the fire, it puffed up and melted.

After these experiments, which were made with the most scrupulous attention, we think that the presence of phosphat of lime in the broth from bones is not at all to be apprehended. However, to leave nothing imperfect with regard to this analysis, we thought it proper to repeat an experiment of Professor Dispan, in order to be finally convinced, whether phosphat of lime be not combined with the broth from bones; but after the jelly had been dried and incinerated, we could not trace the least phosphat of lime. A curious circumstance to be observed is, that broth made from flesh contains phosphat of lime, which has been proved by Professor Dispan; and as this broth is universally found wholesome and nourishing, why should not the broth from bones have the same quality, even if some traces of phosphat of lime were discovered in it?

From this analysis we may justly conclude,

1. That bones contain a great quantity of jelly, sufficient for affording a wholesome and cheap food, and also a small quantity of muriat and of phosphat of soda.

2. That we need not apprehend the presence of phosphat of lime in the broth furnished by bones, because this saline substance cannot be separated from the bones by the common proceeding of decoction.

3. That five pounds of bones have only yielded the same quantity of jelly, though M. Cadet-de-Veaux, in his memoir on the alimentary economy, states, that he has extracted four ounces of jelly from one ounce of bones; which difference, however, much depends on the age of the animals from which the bones are taken, as young bones always yield a greater portion of jelly than old ones.

4. That even if we obtain only the same weight of jelly, great advantages might accrue to all ranks of society by this method of making broth from bones.

5. That all governments ought to encourage this discovery, by ordering such broths to be introduced into hospitals, and similar institutions.

